# The Static Magnetic Field Remotely Boosts the Efficiency of Doxorubicin through Modulating ROS Behaviors

**DOI:** 10.1038/s41598-018-19247-8

**Published:** 2018-01-17

**Authors:** Behnam Hajipour Verdom, Parviz Abdolmaleki, Mehrdad Behmanesh

**Affiliations:** 1 Department of Biophysics, Faculty of Biological Sciences, Tarbiat Modares University (TMU), Tehran, Iran; 2 Department of Genetics, Faculty of Biological Sciences, Tarbiat Modares University (TMU), Tehran, Iran

## Abstract

Exposure to magnetic field (MF) can affect cellular metabolism remotely. Cardio-toxic effects of Doxorubicin (DOXO) have limited clinical uses at high dose. MF due to its effect on reactive oxygen species (ROS) lifetime, may provide a suitable choice to boost the efficacy of this drug at low dose. Here, we investigated the potential effects of homogenous static magnetic field (SMF) on DOXO-induced toxicity and proliferation rate of cancer cells. The results indicated that SMF similar to DOXO decreased the cell viability as well as the proliferation rate of MCF-7 and HFF cells. Moreover, combination of 10 mT SMF and 0.1 µM DOXO decreased the viability and proliferation rate of cancer and normal cells in a synergetic manner. In spite of high a GSH level in cancer cell, SMF boosts the generation and lifetime of ROS at low dose of DOXO, and overcame to GSH mediated drug resistance. The results also confirmed that SMF exposure decreased 50% iron content of cells, which is attributed to iron homeostasis. In conclusion, these findings suggest that SMF can decrease required dose of chemotherapy drugs such as DOXO and thereby decrease their side effect.

## Introduction

Cancer is often initiated by uncontrolled division in a single abnormal cell in different tissues of lung, brain, breast and etc. Especially, breast cancer as the most common malignancy in women leads to many death worldwide annually^1^. However, conventional breast cancer treatment methods like radiation therapy, chemotherapy, surgery and etc. are suffered from high side effects and low efficiency^2^.

Magnetic field (MF) can penetrate into the living organisms and influence their biological and electrobiochemical systems^3^. Static magnetic field (SMF) can directly interact with ions, metals, proteins and some radical pair recombination through well-known physical mechanisms within the cells^4^. It is assumed that SMF exposure can increase the concentration and activity of paramagnetic free radicals in the biological systems^5^. Two major reactive species of free radicals are reactive oxygen species (ROS) and reactive nitrogen species (RNS)^6^. More importantly, *in vitro* and *in vivo* studies have demonstrated that SMF exposure has inhibitory effects on cancer cells^7–9^.

Doxorubicin (Adriamycin), Epirubicin (Ellence), Docetaxel (Taxotere) and Paclitaxel (Taxol) are among the most common types of chemotherapy drugs, which are currently used to treat breast cancer in women^10^. DOXO is a member of anthracycline family that is synthesized by *Streptomyces peucetius*^11^. For decades, DOXO has been considered as a cytotoxic drug for treatment of many cancer types including breast, lung, stomach, bone and thyroid cancers^12^. On the other side, DOXO treatment induces the toxic effects on the patient’s heart, which leads to dose-limited of DOXO use^13^. Previous studies suggest that DOXO-induced cell death might be due to intercalation into DNA, creation of DNA double-strand breaks by inhibition of topoisomerase II, generation of ROS as superoxide anion (O_2_^•−^), hydrogen peroxide (H_2_O_2_) and inhibition of DNA and RNA synthesis^11,14^.

Mammalian cells need iron as an essential factor for crucial metabolic functions such as cellular respiration, electron transfer, ATP production, DNA biosynthesis and etc^15^. In addition, iron catalyzes the production of hydroxyl radical (^•^OH) and increases ROS generation content through Fenton or Haber-Weiss reaction^16^. Therefore, iron is potentially cytotoxic and can induce oxidative stress and DNA damage in cells^17^.

Free radicals are the by-products of cellular metabolism. They act as regulatory mediators in the regulation of cell homeostasis at low or moderate concentrations. In fact, these biomolecules play a crucial role in many fundamental biological processes such as response to environmental stresses, cell signaling, programmed cell death, cell cycle and etc^18,19^. In contrast, free radicals at high concentrations attack to biologically important molecules and lead to oxidative damage of protein, nucleic acids as well as peroxidation of lipids and consequently, activation of cell death pathways^20^.

A big challenge in the chemotherapy is drug resistance, which is affected by different factors^21^. Studies showed that SMF has more effects with chemotherapy drugs such as Adriamycin in the tumor cells^22,23^. It seems that treatments with multiple-agent as used in combination of physical (radiations, MF) and chemical (drugs) agents could increase the antitumor efficacy and decrease the drug-resistance in tumors^24^. We aimed to examine the impact of continuous homogeneous SMF exposure on the facilitation of ROS (^•^OH, O_2_^•−^) generation and on iron content changes as well as its effect on DOXO performance in breast cancer cells.

## Results

### Cell viability experiment upon SMF exposure

**Figure 1:**
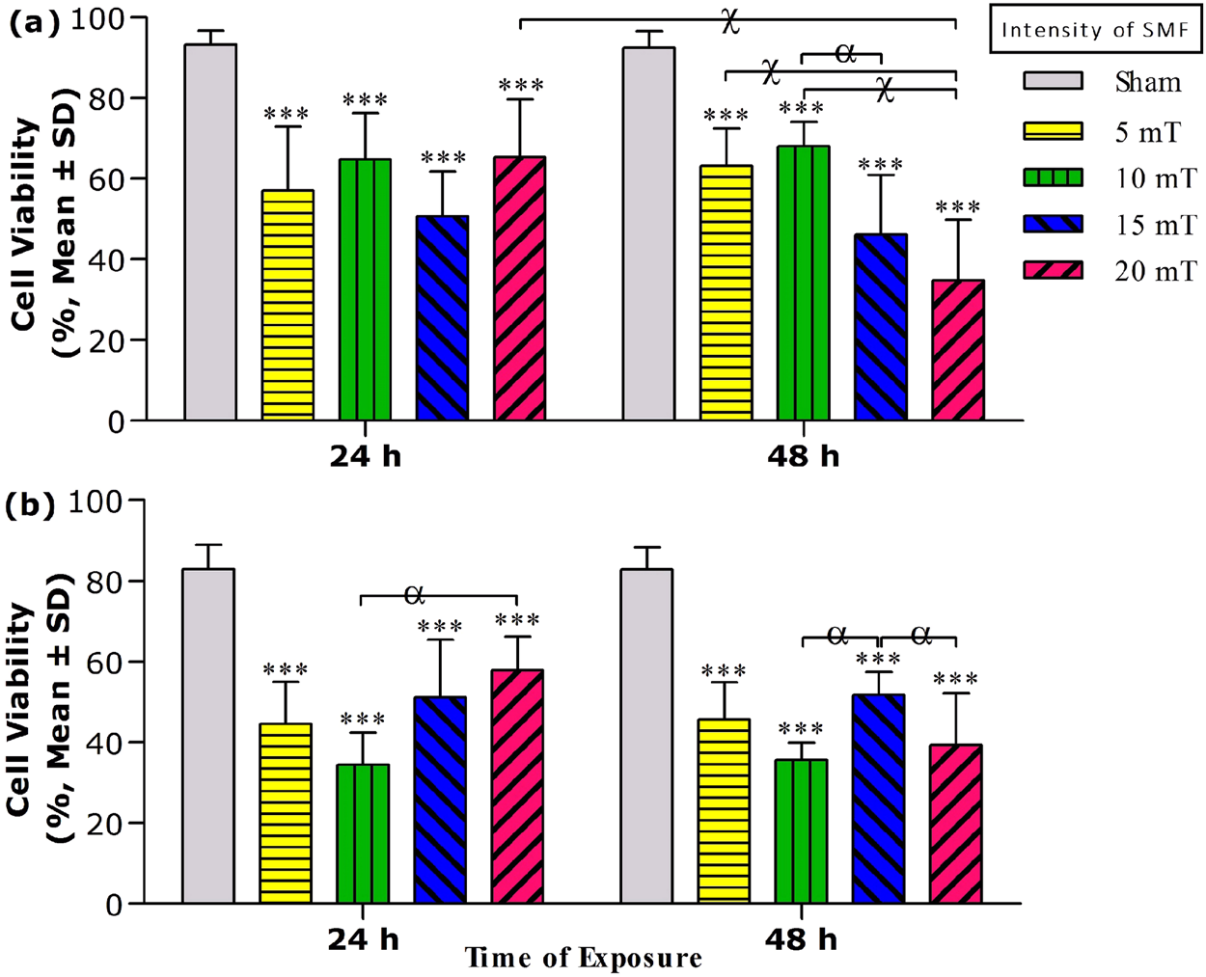
The cell viability results of (**a**) MCF-7 and (**b**) HFF cells exposed with SMF at different intensities (5, 10, 15 and 20 mT) for two exposure times (24 and 48 h). The cell viability value was determined by Trypan blue assay, and results are expressed as the percentage of viable cells. Data are shown as mean ± SD (n = 3). ***P < *0.001*. Significant difference relative to unexposed cells (sham), and letters (*α*,*P* < *0.05*; χ,*P* < *0.001*). Significant differences between treated cells (5 × 2 factorial ANOVA flowed by post-hoc Newman–Keuls multiple comparison tests).

We assessed the effect of continuous SMF exposure on the viability of MCF-7 and HFF cells at different intensities (5, 10, 15 and 20 mT) for both exposure times (24 and 48 h). As shown in Fig. 1a, SMF exposure significantly decreased the viability of MCF-7 cells at both exposure times. Results showed that the viability of MCF-7 cells had no significant effect on exposed cells by the increase of SMF intensity at 24 h. MCF-7 viabilities were decreased to 51 ± 1% after the 15 mT SMF exposure at 24 h as well as 35 ± 15% at 48 h, respectively. The lethal concentration 50% (LC_50_) value of SMF was measured 18 ± 7 and 13 ± 3 mT for MCF-7 cells at 24 and 48 h, respectively. SMF exposure at all intensities also significantly decreased the HFF cell viability compared to unexposed cells at both exposure times (24 and 48 h) (Fig. 1b). Cell viability of HFF cells was also decreased to 35 ± 8% after 10 mT SMF exposure at 24 h as well as 36 ± 4% at 48 h, respectively. The LC_50_ value of SMF was calculated 8 ± 4 and 9 ± 3 mT for HFF cells at 24 and 48 h, respectively. These results indicated that the toxic effects of SMF on normal cells were higher than their effect on MCF-7 as cancer cells.

### Cell viability in the presence of DOXO without magnetic field exposure

**Figure 2:**
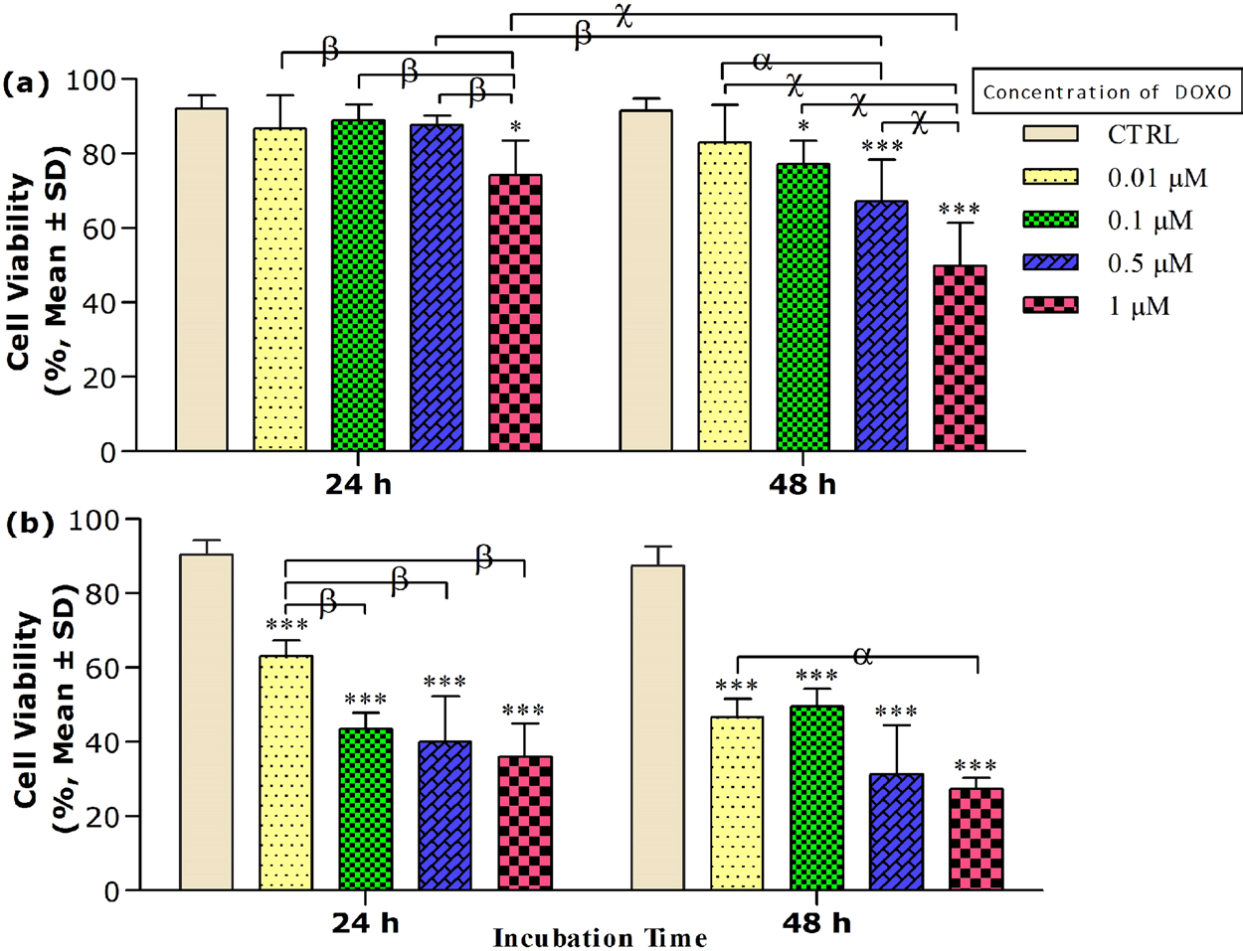
The cell viability results of (**a**) MCF-7 and (**b**) HFF cells treated with DOXO at different concentrations (0.01, 0.1, 0.5 and 1 μM) for two exposure times (24 and 48 h). The cell viability was determined by Trypan blue assay, and results are expressed as the percentage of viable cells. Data are shown as mean ± SD (n = 3). *P < 0.05; ***P < *0.001*. Significant difference relative to untreated cells (CTRL), and letters (*α*, *P* < *0.05*; *β*,*P* < *0.01*; χ,*P* < *0.001*). Significant differences between treated cells (5 × 2 factorial ANOVA flowed by post-hoc Newman–Keuls multiple comparison tests).

The DOXO-induced cytotoxicity at different concentrations (0.01, 0.1, 0.5 and 1 μM) was measured of cells that treated without any MF exposure for both exposure times (24 and 48 h). As shown in Fig. 2a, DOXO significantly decreased MCF-7 viability only in 1 μM at 24 h as well as all concentrations (except 0.01 μM) at 48 h compared to untreated cells, respectively. The viability of MCF-7 cells of DOXO was decreased to 88 ± 9% and 50 ± 11% in 1 μM after 24 and 48 h, respectively. The LC_50_ value of DOXO was measured 3 ± 1 and 0.9 ± 0.2 μM for MCF-7 cells at 24 and 48 h, respectively.

DOXO treatments also decreased the HFF cell viability compared to untreated cells at both exposure times (24 and 48 h) (Fig. 2b). The HFF cell viability of DOXO was decreased to 36 ± 9% and 7 ± 3% in 1 μM concentration after 24 and 48 h, respectively. The LC_50_ value of DOXO on HHF cells was calculated 0.1 ± 0.04 and 0.07 ± 0.02 μM after 24 and 48 h, respectively. Results indicated a descending trend in HFF cell viability the increase of DOXO concentration.

### The proliferation of cells exposed to SMF or DOXO

**Figure 3:**
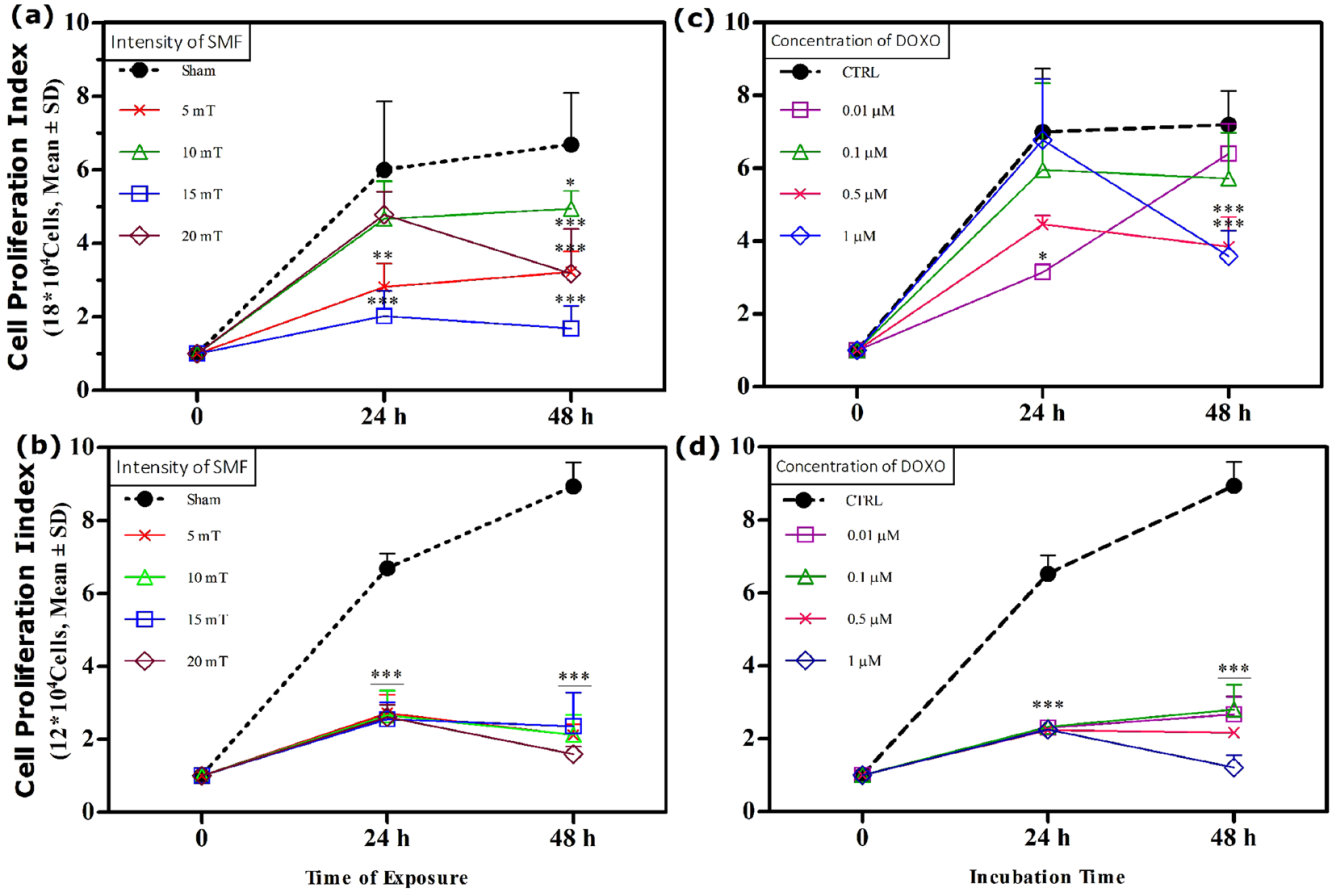
The cell proliferation results of (**a**,**c**) MCF-7 and (**b**,**d**) HFF cells exposed with SMF at different intensities (5, 10, 15 and 20 mT) and incubated with DOXO at different concentrations (0.01, 0.1, 0.5 and 1 μM) for two exposure times (24 and 48 h). The results are expressed as the fold-value of proliferation rate. Data are shown as mean ± SD (n = 3). *P < 0.05; **P < 0.01; ***P < *0.001*. Significant difference relative to unexposed cells (sham, CTRL) (***; refers to all cell groups) (5 × 2 factorial ANOVA flowed by post-hoc Newman–Keuls multiple comparison tests).

We investigated the proliferation rate of MCF-7 and HFF cells in presence of SMF and DOXO. We found that SMF significantly decreased number of MCF-7 cells after exposure to 5mT and 15 mT at 24 h, as well as 5–20 mT at 48 h compared to unexposed cells (Fig. 3a). In presence of 5 and 15 mT SMF exposure, the proliferation rate of cells was decreased to 3.2 and 3.9-fold compared to unexposed cells (proliferation rate: 6-fold) at 24 h, respectively. In addition, the proliferation rate of MCF-7 was decreased to 3.5, 5 and 3.5-fold after the exposure to the 5 mT, 15 mT and 20 mT, respectively compared to unexposed cells (proliferation rate: 6.7-fold) at 48 h.

The proliferation rate of HFF cells was also significantly decreased in presence of SMF intensities compared to unexposed cells at 24 and 48 h (Fig. 3b). Results indicated that SMF exposure similarly decreased the proliferation rates of HFF cells circa (ca.) 4.1-fold compared to unexposed cells (proliferation rate: 6.7-fold) at 24 h, as well as ca. (6.6–7.4)-fold compared to unexposed cells (proliferation rate: 8.9-fold) at 48 h.

DOXO significantly decreased the proliferation rate of MCF-7 cells in treatments of 0.01 μM at 24 h, as well as 0.5 μM 1 μM at 48 h compared to untreated cells (Fig. 3c). The proliferation rate of cells was decreased to 3.9-fold in the DOXO treatment (0.01 μM) compared to untreated cells (proliferation rate: 7-fold) at 24 h. In addition, proliferation rate was decreased to 3.4-fold and 3.6-fold in treatments of 0.5 μM and 1 μM DOXO compared to untreated cells (proliferation rate: 7.2-fold) at 48 h, respectively.

DOXO significantly decreased the proliferation rate of HFF cells in all concentrations compared to untreated cells at both exposure times (Fig. 3d). The proliferation rates were decreased ca. 4.3-fold compared to untreated cells (proliferation rate: 6.5-fold) at 24 h and ca. (6.1–7.7)-fold compared to untreated cells (proliferation rate: 8.9-fold) at 48 h.

### The SMF and DOXO affects the intracellular concentration of iron

**Figure 4:**
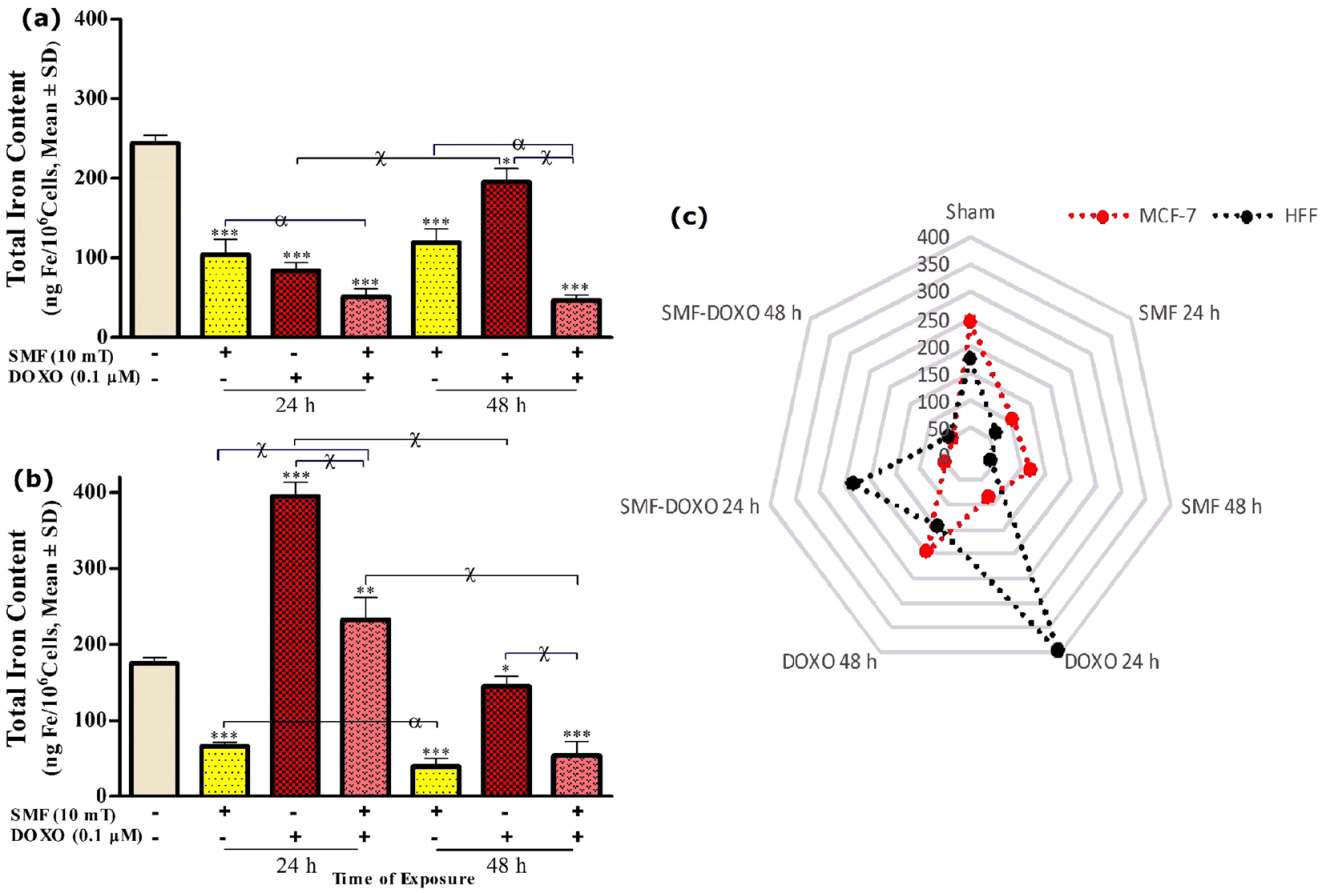
The intracellular iron results of (**a**) MCF-7, (**b**) HFF cells and (**c**) both comparison exposed with 10 mT SMF and incubated with 0.1 μM DOXO for two exposure times (24 and 48 h). The total iron was quantified by ICP-OES, and results are expressed as the concentration of iron content (ng Fe per 10^6^ cells). Data are shown as mean ± SD (n = 3). *P < 0.05; **P < 0.01; ***P < *0.001*. Significant difference relative to unexposed cells (sham; was measured at 48 h), and letters *(α*,*P* < *0.05;* x,*P* < *0.001)*. Significant differences between treated cells (2 × 2 × 2 factorial ANOVA flowed by post-hoc Newman–Keuls multiple comparison tests).

ICP-OES data analysis of MCF-7 cells showed that total intracellular concentration of iron (Fe (II) and Fe (III)) was significantly decreased in the presence of 10 mT SMF, 0.01 μM DOXO and combined treatments (Fig. 4a). The iron content of MCF-7 cells was 104.2 and 119.2 (ng Fe/10^6^ cells) in SMF exposure, 83.7 and 195.3 (ng Fe/10^6^ cells) in DOXO treatment, 50.9 and 46.9 (ng Fe/10^6^ cells) in combined treatments compared to unexposed cells (247 ng Fe/10^6^ cells) at 24 and 48 h, respectively. DOXO treatment showed the increase of cellular iron content at 48 h. A significant decrease was observed in the iron content of MCF-7 cells in both treatments (SMF and DOXO). The SMF and DOXO significantly altered the intracellular iron content of HFF cells (Fig. 4b). The Iron content of HFF cells was 65.6 and 30.3 (ng Fe/10^6^ cells) in SMF exposure, 395.2 and 144.9 (ng Fe/10^6^ cells) in DOXO treatment, 232.2 and 53.9 (ng Fe/10^6^ cells) in the presence of both treatments compared to unexposed cells (175 ng Fe/10^6^ cells) at 24 and 48 h, respectively. In HFF cells, combined treatments (SMF and DOXO), as well as DOXO, caused an early increase in the iron content at 24 h and then a decrease at 48 h. The total iron content of MCF-7 was more than HFF (P < 0.01) in the unexposed cells (Fig. 4c).

### The effects of SMF and DOXO on intracellular ROS generation

**Figure 5:**
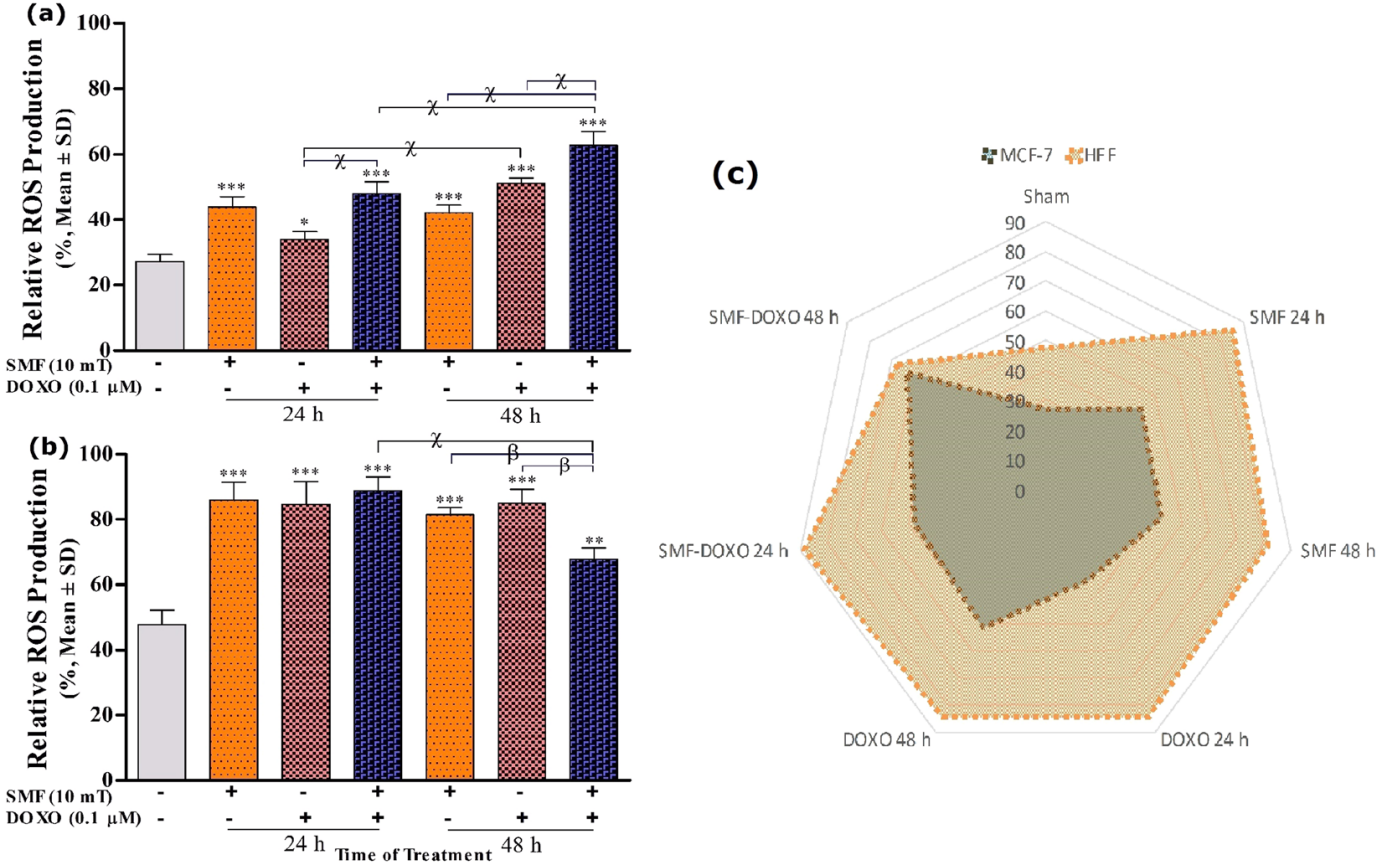
The cellular ROS results of (**a**) MCF-7, (**b**) HFF cells and (**c**) both comparison exposed with 10 mT SMF and incubated with 0.1 μM DOXO for two exposure times (24 and 48 h). The ROS production was measured by flow-cytometry fluorescent probe DCFDA, and results are expressed as the percentage of ROS production (fluorescent intensity per 10^4^ cells). Data are shown as mean ± SD (n = 3). *P < 0.05; **P < 0.01; ***P < *0.001*. Significant difference relative to unexposed cells (sham; was measured at 48 h), and letters *(β*,*P* < *0.01;* X,*P* < *0.001)*. Significant differences between treated cells (2 × 2 × 2 factorial ANOVA flowed by post-hoc Newman–Keuls multiple comparison tests).

The ROS concentration of MCF-7 cells increased significantly in the presence of 10 mT SMF, 0.1 μM DOXO and combined both treatments (Fig. 5a). ROS content of MCF-7 cells was 43.91 and 42.31% in SMF exposure, 33.90 and 51.23% in DOXO treatment, 48.04 and 62.80% in the presence of both treatments compared to unexposed cells (27.2%) at 24 and 48 h, respectively. Combined DOXO treatment showed the increase of ROS levels at 48 h. treatments (SMF and DOXO) showed a significantly higher ROS content MCF-7 cells than a single treatment. We found that intracellular ROS production significantly increased in HFF cells (Fig. 5b). ROS content of HFF cells was 86 and 81.56% in SMF exposure, 84.78 and 85.14% in DOXO treatment, 88.83% and 67.84% in the presence of both treatments compared to unexposed cells (47.7%) at 24 and 48 h, respectively. In the HFF cells, combination treatments (SMF and DOXO) caused an early increase in the ROS level at 24 h and then a decrease at 48 h. The maximum fold-change values in ROS content were approximately 2.3-fold and 1.8-fold in MCF-7 and HFF cells treatments, respectively. However, the ROS concentration of unexposed cells was in the MCF-7 more than HFF (P < 0.001) (Fig. 5c). The effects of SMF or DOXO treatments on MCF-7 cells was more than those of HFF cells.

### Glutathione content status of cells exposed to SMF and DOXO

**Figure 6:**
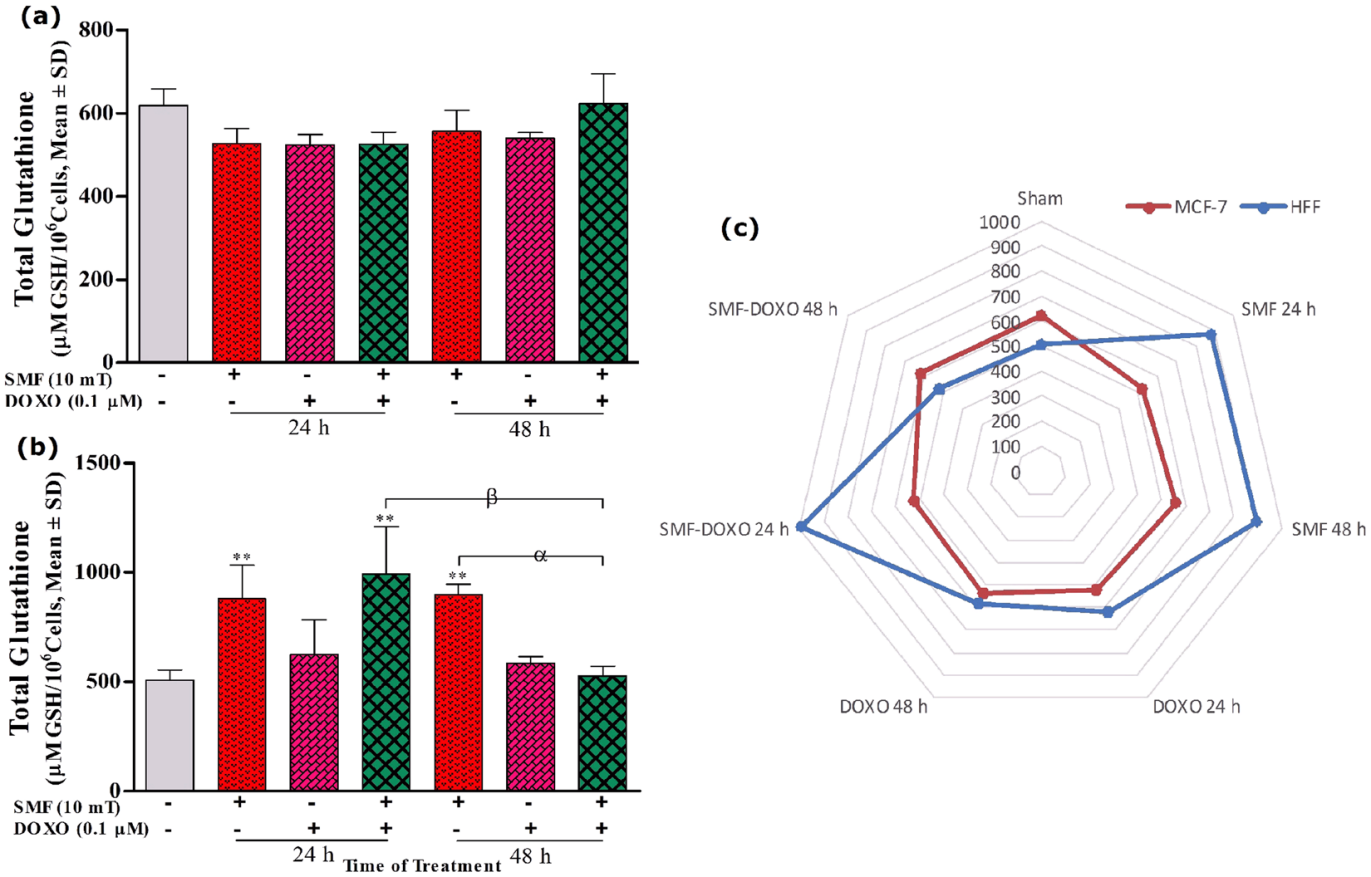
The total intracellular glutathione (tGSH) results of (**a**) MCF-7, (**b**) HFF cells and (**c**) both comparison exposed with 10 mT SMF and incubated with 0.1 μM DOXO for two exposure times (24 and 48 h). The GSH was measured by a microplate reader, and results are expressed as the concentration of glutathione content (μM GSH per 10^6^ cells). Data are shown as mean ± SD (n = 3). **P < 0.01. Significant difference relative to unexposed cells (sham; was measured at 48 h), and letters (α,P < 0.05; β,P < 0.01). Significant differences between treated cells (2 × 2 × 2 factorial ANOVA flowed by post-hoc Newman–Keuls multiple comparison tests).

The level of total intracellular glutathione (GSH) of MCF-7 cells was determined following treatment with 10 mT SMF, 0.1 μM DOXO and combination treatments. As shown in Fig. 6a, none of treatment caused to a significant change in GSH level of MCF-7 cells. Interestingly the MCF-7 cells should a significantly higher GSH level compared to HFF cells. However, the GSH content of HFF cells showed significant increase in the 10 mT SMF exposure and combined treatment (Fig. 6b). In presence of either SMF or DOXO, the GSH level of HFF cells show an intense increment of GSH followed by significant decrement at 48 h.

## Discussion

In this study, we have mainly focused on synergistic cytotoxic effects of homogeneous SMF and DOXO on normal (e.g., HFF) and cancer cells (e.g., MCF-7). Cellular behavior in mammalian tissue is controlled by microenvironment, which includes extracellular matrix, blood vasculature and various stromal cell types (e.g., endothelial cells, fibroblasts, and myofibroblasts). Especially, in breast carcinomas, epithelial-derived tumor cells (e.g., MCF-7) is surrounded by fibroblasts, which influences on their morphology and behaviors (initiation and progression)^25–28^. For this reason, we used stromal-derived fibroblast (HFF) as a normal cell, which has same embryonic origins of mesoderm layer with mammary gland fibroblasts^29–31^.

Here, SMF exposure increased the DOXO efficiency by stimulating ROS production, while alone SMF exposure did not induce high-level cell toxicity. In another study, SMF increased the effective of other chemotherapy agent like 5-FU and Taxol^23^.

SMF has various intensity ranges, weak (<1 mT), moderate (1 mT-1 T) and high (1 T<)^3^. MF modulate many cellular structures and functions such as membranes, mitotic spindle, ion concentration, gene expression, cell cycle, proliferation and differentiation^32–34^. Recent studies demonstrated that MF increases the permeability of the cellular membrane, which in turn enhance the intracellular Ca^2+^ concentration. This leads to the increase of cell viability and ROS content and the decrease of cells apoptosis^35–37^. There are collection of controversial reports about the effects of SMF on cancer cells. Some *in vitro* and *in vivo* studies indicated that SMF has little toxic effects on tumor cells^32^. In contrast, other studies have shown that cancer cells are very sensitive to SMF^22,38^. Our results indicated that SMF could decrease the cell viability and proliferation rate of MCF-7 and HFF cells (Figs 1, 3a,b). Moreover, MF caused to oxidative damage of nucleic acid and proteins and overwhelmingly increased the risk factor for cancer occurrence in the normal cells^3,39^. It was found that being to SMF, which produced by occupational exposure (such as aluminum and chloralkali industries) increase the occurrence of leukemia, brain and breast cancers^40,41^. Several mechanisms have been proposed to relate MF with chemical changes, which occurs within the cells. MF influence the biological systems through biophysical and biochemical interactions such as Fenton and Haber-Weiss reactions, which can finally produce ^•^OH as the most dangerous and cytotoxic free radical^5,16,42^.

DOXO can trigger apoptotic pathways through mechano-chemically damages, which lead to the death of tumor cells^14^. However, cancer cells use different drug-resistance strategies to evade apoptosis and intern reduce the efficacy of chemotherapic agent like DOXO^43,44^. Cellular uptake of DOXO is influenced by human epidermal growth factor receptor-2 (HER2) expression. DOXO highly impacts on HER2-positive tumor cells with overexpress HER2 gene^45^. MCF-7 cells are HER2-negative, thus have low penetration of DOXO and moreover, have very powerful mechanisms to repair the cellular damages that show chemo-resistance in regard to DOXO^46,47^. Our results showed that DOXO decreased the cellular viability and proliferation rate of MCF-7 cells (Figs 2a, 3c), which were more susceptible at higher concentrations and long incubation times. In contrast, HFF cells show a high sensitivity to DOXO treatment (Figs 2b, 3d). However, we expected that our cancer cells be sensitive to either DOXO or SMF because tumor cells have high metabolic activities^48^. DOXO has more toxic effects on normal cells. Based on LC_50_ measurement, we found that HFF cells were very sensitive to SMF and DOXO. MCF-7 showed more tolerance behaviors in the presence of these treatments (Figs 1, 2, 3).

DOXO activation occurs in presence of one-electron redox-cycling reaction, which leads to the production of DOXO-semiquinone, superoxide and hydrogen peroxide. Indeed, DOXO receives one electron from interaction of O_2_ with intracellular iron accumulation and finally, Fe (II) is released from ferritin^11,49^.

Iron is critical for cellular functions such as metabolism, growth, and replication. Iron also participate in mitochondrial enzymes, DNA synthesis and repair, signaling pathways and metabolic detoxification such as peroxidase and catalase^50^. There is a relationship between iron storage, cancer risk, and tumor growth^51^. Tumor cells absorb Fe-ions from surrounding normal cells by the dysregulation of iron homeostasis and abnormal modifications of iron metabolism, and iron storage in form of various complexes such as iron-sulfur (Fe-S) clusters^15,50^.

Normal cells usually export extra intracellular iron. In contrast, tumor cells induce the overexpression of iron-regulatory proteins that contribute to iron absorption and metabolism^50,52^. This process is known as iron stolen. As many studies have indicated cancer patients often suffer from iron deficiency and anaemia^53^.

Here, we further examined the total intracellular iron concentration of cells, treated with 10 mT SMF and 0.1 µM DOXO. The results showed that MCF-7 iron storage was higher than of HFF cells (Fig. 4c). Similarly, simultaneous treatments of cells with SMF and DOXO drastically decreased the iron content of tumor cells compared to untreated cells (Fig. 4a). Although variations of HFF iron content did not show a similar trend with MCF-7 cells. it was related to incubation times (Fig. 4b). Moreover, MCF-7 cells in the presence of either DOXO or SMF, try to export some part of their iron content. However, non-tumor cells cannot export extra iron, which in turn leads to their death (Figs 1b, 2b, 4b). Due to deleterious and cytotoxicity effects, iron is used to design chelators for hyperthermia in cancer therapy^54^.

The increase of iron concentration is critical for the production of free radical through Fenton reaction in cells^16^. Low and moderate concentrations of intracellular free radicals contribute to the regulation of homeostasis and critical processes^18,19^, while at high concentrations, induce severe stresses such as oxidative damage to DNA, proteins, and membranes^20,55^. ROS can also cause DNA adducts and interstrand cross-links as well as DNA base modification and strand breaks^56^.

In the present study, we assessed the ROS production in the presence of 10 mT SMF and 0.1 µM DOXO. We found that ROS content in HFF cells was ca. 1.7-fold more than that of MCF-7 cells. Compared to unexposed cells, the concentration of DCF-detectable ROS showed a significant increase ca. 2-fold in MCF-7 and HFF cells exposed to SMF or DOXO (Fig. 5).

Our data suggested that combined treatments cause high ROS production in MCF-7 cells over time (Fig. 5a). However, combined treatments in HFF cells show higher ROS content at 24 h than 48 h (Fig. 5b). To explain the exact mechanism of SMF effects on the production of free radicals, it can be suggested that it provides the energy required to excite the atomic external electrons or the energy required for the interaction between the electron spins and nucleus^5^.

Radical pairs have unpaired electron spins either antiparallel (with zero total electron spins, ↑↓, singlet state, S) or parallel (with same spins, ↑↑, triplet state, T). Unlike S-state, T-state has an associated magnetic moment more than zero^57^. MF can impact on the spin states of radical pairs and the interconversion and chemical fates of S and T energy states. Energy levels of T-state can align and split by MF and finally induce the production of free radicals. This occurs in a well-known interaction known as Zeeman Effect^5,57^. MF influence on the longevity and chemical function of free radicals through exciting T energy state, so that it increases ^•^OH lifetime from one nanosecond to one second^5,58^. Although there are several experimental studies indicating that MF can produce free radicals in the cells, most studies are about the production of free radicals based on the concepts of theoretical physics^59,60^.

Tumor cells have abnormal metabolic activities which lead to ROS production^56,61^. However, in this study, we have revealed that ROS content in tumor cells is less than normal cells (Fig. 5c). Cancer cells are able to make greater responses to oxidative stresses, for example, they can tolerate ROS by changing the redox homeostasis. Cancer cells can produce two major antioxidant molecules namely nicotinamide adenine dinucleotide phosphate (NADPH) and glutathione (G-SH) extensively, like scavengers, which protect cells against ROS-mediated toxicities^48^. Here, we determined the total GSH concentration of cells. The results showed that GSH content of MCF-7 was higher than that of HFF cells (Fig. 6c). Simultaneous treatments with SMF and DOXO did not show a significant difference in the GSH content of tumor cells compared to unexposed cells (Fig. 6a). The HFF cells were showed increase of GSH cellular concentration in the SMF exposure compared to unexposed cells (Fig. 6b). Cancer cells have delicate mechanisms to regulate intracellular ROS generation^48^. According to our results, MCF-7 cells are more responsive and resistant to cellular stresses such as SMF and DOXO than HFF cells.

**Figure 7:**
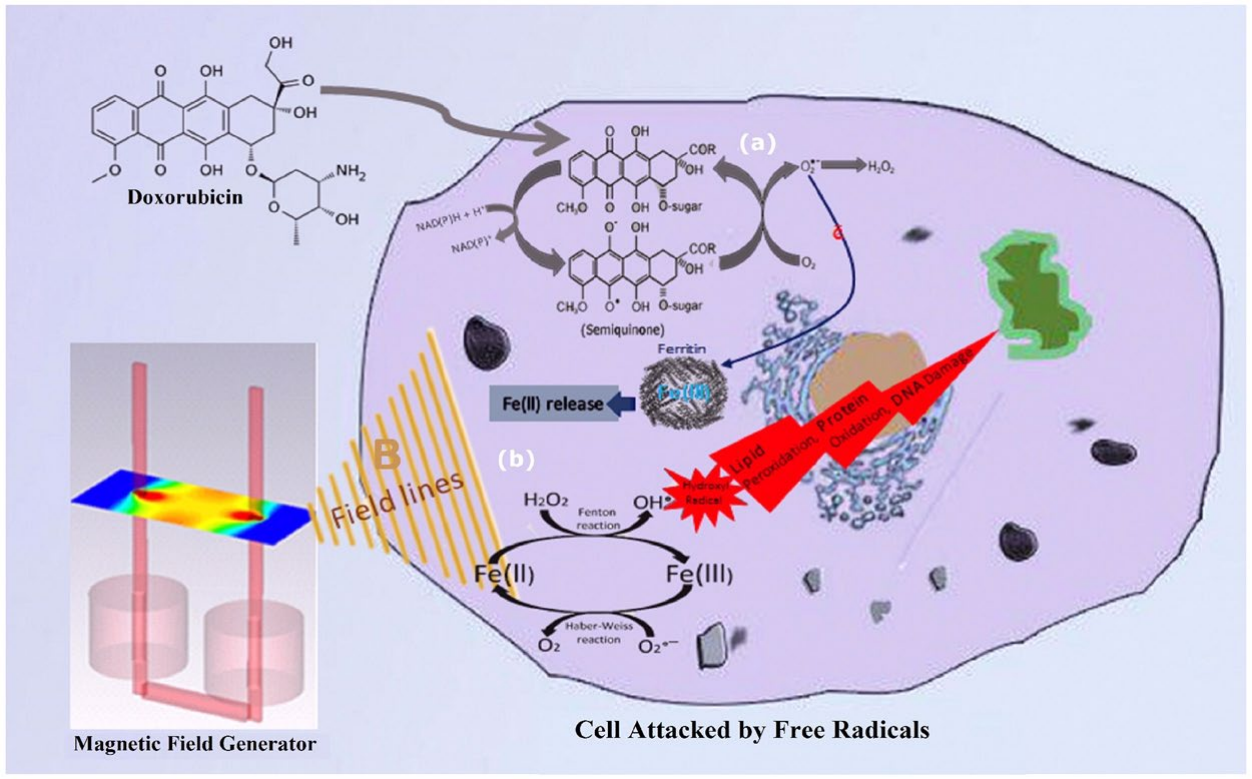
Schematic diagram of intracellular cross-talk in ROS generation by homogeneous magnetic field and Doxorubicin. (**a**) Doxorubicin induces Fe (II) release by direct interactions of O_2_^•−^ with intracellular iron accumulations as ferritin, (**b**) in parallel magnetic field amplifies formation of ROS through Fenton reaction, which lead to DNA damage, protein oxidation and lipid peroxidation.

Our findings indicated that combination of SMF and DOXO increase the efficiency of DOXO against cancer cells (Fig. 7). However, this combination showed the higher toxic effect on normal cell compared to cancer cell, presumably due to acquired drug resistance abilities (such as DNA damage repair, drug efflux pumps, antioxidant systems and etc.) of cancer cells^62^. Fortunately, progress during last decade researchers has developed carriers, which specifically deliver their cargo to cancer cells and prevent uptake by normal cells. Therefore, it is possible to reduce the side effect of this combination by using the targeted delivery formulation of DOXO and local exposure of magnetic fields on tumor tissue^63–66^. Moreover, Photosensitizer produced free radicals as singlet oxygen (^1^O_2_) in order to damage or killed the tumor cells^67^. Therefore, MF may be considered as a co-photosensitized agent in local exposure which may improve the efficiency of Photodynamic therapy (PDT).

## Conclusion

This study aimed to determine the effects of continuous homogenous SMF in combination with DOXO on normal and tumor cells, and to illuminate the mechanism behind this effects. The results of this study confirmed that combination of SMF and DOXO efficiency decreased the cell viability and hampered the proliferation of cells. Analysis of ROS generation, iron, and GSH contents reveal an interesting mechanism. Briefly, DOXO-mediated ROS generation facilitate release of iron from accumulation. In next step, the released iron incorporated in SMF catalyzed Fenton reaction, while produce addition ROS for induction of cell death. In the other hand, SMF or DOXO as two sources of stress, deregulated the metabolism and homeostasis of intracellular iron. In response to the stress, cells export iron to decrease their iron content. Therefore, inhibition of iron exertion, may improve the efficiency of this combination. Consequently, the results suggest that co-treatment of MF and DOXO can kill the cancer cells synergistically by increasing free radicals. Therefore, MF present promising ways to improve therapeutic methods with ROS-dependent drugs such as Photo-chemotherapeutics.

## Materials and Methods

### Chemical reagents

Dulbecco’s Modified Eagle’s Medium (DMEM) and Fetal Bovine Serum (FBS) were purchased from Gibco. Penicillin-streptomycin and trypsin-EDTA were obtained from Bioidea. Doxorubicin was obtained from Accord Healthcare. 2′,7′-dichlorofluorescein diacetate (DCFDA) (ab113851) was purchased from Abcam. Glutathione (GSH) assay kit (ZB-GSH-48A) was purchased from ZellBio GmbH, Germany. Trypan blue, HCl and HNO_3_ were obtained from Merck.

### Cell culture

The human breast adenocarcinoma cell line (MCF-7) and human foreskin fibroblast (HFF) cells were used to study. They were obtained from the National Cell Bank of Iran (NCBI) and Pasteur Institute of Iran (IPI). The cells were allowed to grow in Dulbecco’s Modified Eagle’s Medium (DMEM) in the neutral PH (7.2–7.4) supplemented with 10% (v/v) heat-inactivated (50 °C, 30 min) Fetal Bovine Serum (FBS) and 2 mM glutamine, 100 units/mL of penicillin and 100 mg/mL of streptomycin at 37 °C and 5% CO_2_ in a humidified incubator (as control condition). The cells were then trypsinized (0.025% trypsin, 0.02% EDTA) after they were grown until 70–80% confluent. Prior to treatments, cells were allowed to reattach to the bottom of the cell culture plate for 24 h.

### Cell proliferation and viability experiments

Cytotoxic effects of the static magnetic field at 5, 10, 15 and 20 mT intensities, Doxorubicin at 0.01, 0.1, 0.5 and 1 μM concentrations and unexposed cells (either sham or control) were determined against MCF-7 and HFF cells using Trypan blue assay. Briefly, MCF-7 (18 × 10^4^ cells/well) and HFF (12 × 10^4^ cells/well) were seeded into 6-well culture plate (SPL Life Sciences Co., Ltd. Korea) and incubated in a total volume of 2 mL supplemented DMEM at 37 °C and 5% CO_2_. Cells were initially allowed to attach overnight and then washed with 1X PBS (PH = 7.2–7.4). The washed cells were prepared in fresh supplemented DMEM with 10% FBS and then were treated with different SMF intensities and DOXO concentrations at both exposure times (24 and 48 h). In Trypan blue exclusion test, cells suspended in PBS were stained with 10% (v/v) Trypan blue for 5 min and analyzed using an inverted microscope (Motic, AE31). The LC_50_ (Medium Lethal Concentration) values characterize the concentration of a substance required to kill 50% of organisms exposed to it in any types of toxicity test^68^. The percentage of cell viability was calculated using the standard formula as shown in Eq. (1).

1 $${\rm{V}}{\rm{i}}{\rm{a}}{\rm{b}}{\rm{l}}{\rm{e}}\,{\rm{c}}{\rm{e}}{\rm{l}}{\rm{l}}{\rm{s}}\,({\rm{ \% }})=({\rm{L}}{\rm{i}}{\rm{v}}{\rm{e}}\,{\rm{c}}{\rm{e}}{\rm{l}}{\rm{l}}\,{\rm{c}}{\rm{o}}{\rm{u}}{\rm{n}}{\rm{t}}\,{\rm{p}}{\rm{e}}{\rm{r}}\,{\rm{m}}{\rm{l}}/{\rm{T}}{\rm{o}}{\rm{t}}{\rm{a}}{\rm{l}}\,{\rm{c}}{\rm{e}}{\rm{l}}{\rm{l}}\,{\rm{c}}{\rm{o}}{\rm{u}}{\rm{n}}{\rm{t}}\,{\rm{p}}{\rm{e}}{\rm{r}}\,{\rm{m}}{\rm{l}})\times 100$$

### Cell-culture magnetic field exposure system

**Figure 8:**
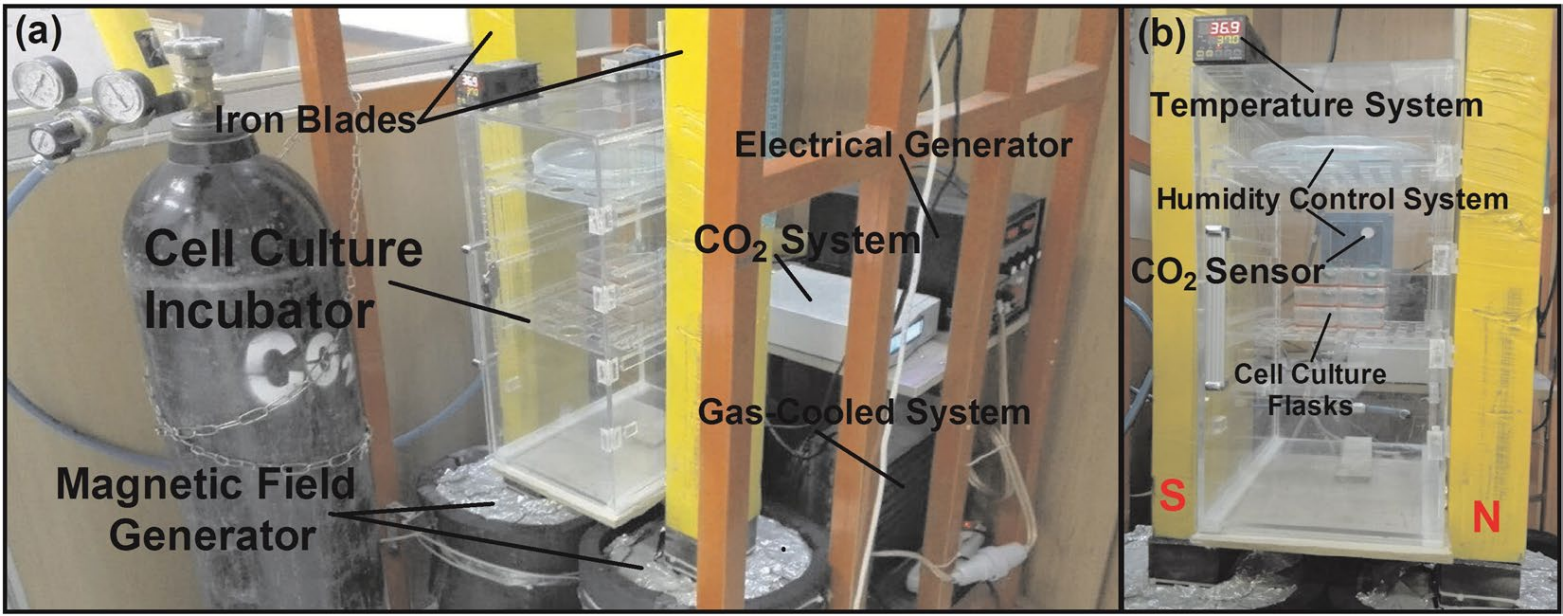
Illustration of magnetic field exposure system. (**a**) The whole body of exposure system. (**b**) The movable plexiglass incubator (exposure unit) within iron blades for providing standard conditions of cell culture.

Exposure to MF was done using a locally designed homogeneous SMF generator. The MF generator consisted of two coils direct current (DC) switching power supply (Fig. 8a). The coils were made of a wire (3.0 mm diameter) and were resistant to heat up to 200 °C. Wirelength in each coil was about 1 km and each coil weighed approximately 40 kg. Coils had a total resistance and inductance of 3 Ω and 2 Henry, respectively. These two coils guided MF through two iron blades (with 1-meter height and a cross-section of 10 × 10 cm^2^). As shown in Fig. 8b, a removable incubator (a plexiglass container with 35 cm length, 23 cm Width and 52 cm Height) as exposure unit was included between both iron blades (with 1 cm gap), which were stabled by plastic bases on wooden insulation (with 1 cm thickness) and also exposure unit is above the coils with circa 15 cm distance. In exposure unit temperature (37 °C), CO_2_ pressure (5%) and humidity were controlled using three different sensors. Cells were exposed to either sham or MF condition.

The electric power was provided using a 220 V/AC power supply equipped with a variable transformer as well as a single-phase full-wave rectifier. The coil received DC voltage up to 50 V and a current up to 16 A form switching power supply and the power provided was equal to 800 W. The effects of current-induced heating were not observed in the exposure unit. Temperature was measured in the inside and outside of incubator, and on the external of coils by thermometer at sham and exposure condition at different currents (Supplementary Fig. S1). To cool off the system, a gas chiller with optimum control on temperature was used. This automatic cooling system consisted of an engine, an evaporator, a condenser and a refrigerant gas. The engine is far enough to exposure unit to avoid any effective MF interference. The evaporator covered the outer surface of the coils, which enabled it to effectively cool the system down. Due to the volume of the operator used in this system, a one-third Danfoss motor was used. The condenser should be one-third as equal to the motor. The gas introduced to this motor was Freon-12 kind (Fig. 8).

**Figure 9:**
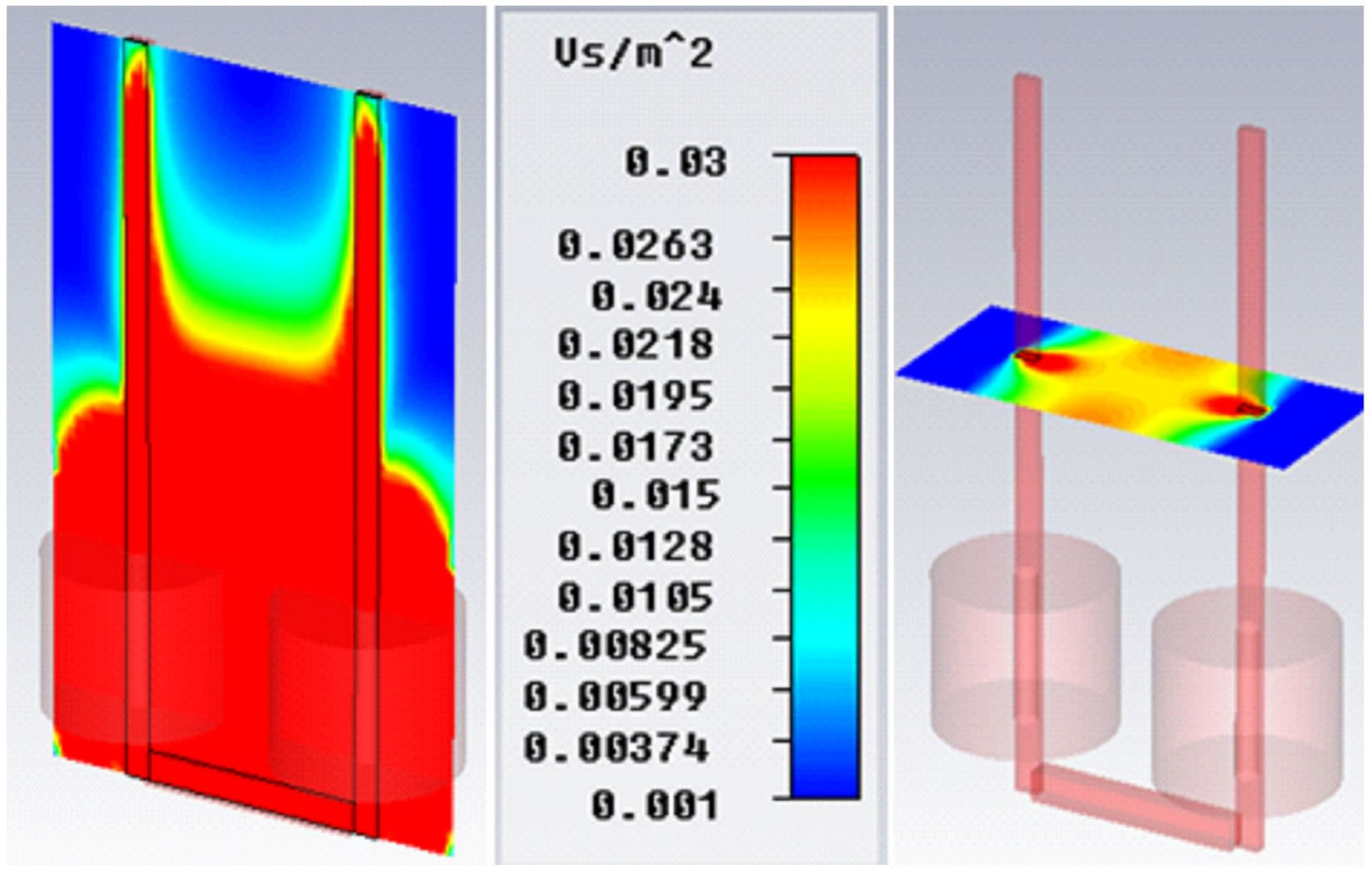
The distribution of magnetic field diagram in the iron blades and coils of exposure system. The place between two iron blades (pink axels), which is highlighted in yellow shows homogeneous magnetic field intensity.

The system was designed to generate homogeneous SMF in the range of 0.5 mT to 90 mT in the stable conditions. An electronic board was used to stabilize the exposure system so that a homogeneous SMF was obtained inside the exposure unit. In addition, a Teslameter (13610.93, PHYWE, Gottingen, Germany) with a probe type of Hall Effect was used to measure MF between iron blades, calibrate the system and test the accuracy and uniformity of SMF. A Hall Effect sensor is a transducer with the varying output voltage in response to MF. An oscilloscope (40 MHz, model 8040, Leader Electronics Co., Yokohama, Japan) was used to test the presence of any pulsation in the current resulted from the rectifier into SMF generating apparatus. This pulsation frequency may be related to the shortcoming of single-phase full-wave rectifier used in the experiment, which provided a ripple voltage around (5%). However, the ripple voltage is not zero, it is small enough to confirm that the generated MF is highly homogeneous. As shown in Fig. 9, MF is calculated by Complete Technology for 3D Simulation (CST STUDIO 2011 software) to select the best site for our samples within the exposure unit of SMF-exposure system (http://www.CST.com). The profile of field emission in coils is demonstrated with surfaces of the same color. The value of geomagnetic field in our lab was 47 µM based on the measurement performed by Tehran geomagnetic observatory, Institute of Geophysics, University of Tehran.

### Quantitation of intracellular iron

To determine the iron content of cells, MCF-7 and HFF cells were seeded in the T-25 cm^2^ culture flask (SPL Life Sciences Co., Ltd. Korea) and allowed to achieve 30–40% confluence. Cells were prepared in DMEM media supplemented with 10% (v/v) heat-inactivated FBS and subsequently were incubated to either sham (was measured after 48 h) or 10 mT SMF, 0.1 μM DOXO and their combination at both exposure times (24 and 48 h). Then cells were washed three times with 1X PBS buffer to remove the extracellular iron ions. The cells were trypsinized and the pellet was dissolved with 1-ml aqua regia (HCl:HNO_3_, 3:1)^69^. No further iron ions are added to cell culture medium. The intracellular iron content of samples was analyzed using an inductively coupled plasma (ICP-OES) spectrometer (Optima 7300 DV, Perkin-Elmer, USA). Data were collected from at least 10^6^ cells.

### Quantitation of intracellular ROS production

Intracellular ROS levels under normal and stress conditions were detected using 2′,7′–dichlorofluorescein diacetate (DCFDA) assay kit. DCFDA is a fluorogenic dye that measures ^•^OH, peroxyl (^•^O_2_H) and other intracellular ROS activities. The following diffusion into the cell, DCFDA was deacetylated by esterase to a non-fluorescent compound that was polar and trapped and subsequently was oxidized by intracellular ROS into 2′,7′–dichlorofluorescein (DCF)^70^. DCF is highly fluorescent and can be detected using fluorescence spectroscopy at 485 nm excitation and 535 nm emission.

MCF-7 and HFF cells (30–40% confluence) were exposed to either sham (was measured after 48 h) or DOXO (0.1 μM) and SMF (10 mT) as well as their combination at both exposure times (24 and 48 h), which were prepared with supplemented DMEM media 10% FBS in the T-25 cm^2^ culture flask. The cells were prepared immediately after treatment as recommended by the manufacturer. Briefly, the cells were washed with 1X   PBS. Then the samples were suspended in the conical test tube with 20 µM DCFDA in the supplemented buffer (10% FBS in the buffer 1X) and incubated for 30–45 min at 37 °C in the dark. Measurement of ROS production was monitored immediately by FACScalibur Becton-Dickinson flow cytometry (Franklin Lakes, NJ). Data were collected from at least 10^4^ cells. The DCFDA flow cytometric data were analyzed by *flowingsoftware* (version 2.5.1, http://www.flowingsoftware.com) with FITC parameters.

### Quantitation of cellular glutathione

Total intracellular glutathione (tGSH) of MCF-7 and HFF cells was measured by using the glutathione (GSH) assay kit (ZellBio GmbH). This kit is based on colorimetric method for analyzing either total GSH or the reduced form GSH alone using a microtiter plate reader. Briefly, MCF-7 and HFF cells were seeded in T-25 cm^2^ culture flask in DMEM media supplemented with 10% (v/v) heat-inactivated FBS and 2 mM glutamine, 100 units/mL of penicillin and 100 mg/mL of streptomycin. The cells were treated with 10 mT SMF exposure, 0.1 μM DOXO and their combination at both exposure times (24 and 48 h). The assay was performed according to protocol provided by the company. Briefly, the cells were washed with 1X PBS buffer and trypsinized. Then 10^6^ cells of each treatment group were lysed through repeated freeze-thaw cycles and centrifuged at 3000 rpm for 20 min. Supernatants were carefully collected. After that 80 μL supernatants were mixed with 20 μL R_2_ buffer (precipitating agent) and centrifuged at 5000 rpm for 10 min. Then 10 μL protein-free supernatants were transferred to the microplate. R_3_ buffer as chromogen (200 μL) was mixed to all wells and incubated 5 min at room temperature. The sham condition was also measured after 48 h. Absorbance was measured by a microplate reader (ELx800, Biotek, USA) at 412 nm. After that, GSH level was calculated based on GSH standard curve and Eq. (2). Standard curve was provided by dissolving GSH into R_1_ buffer 1X (assay buffer) for preparation of 1000 μM GSH.

2 $$GSH(\frac{\mu mol}{L})=\frac{(\mathrm{OD}\,\mathrm{sample}-\mathrm{OD}\,\mathrm{blank})}{(\mathrm{OD}\,\mathrm{standard}-\mathrm{OD}\,\mathrm{blank})}\times 1000\,\mu mol/L$$

### Statistical analysis

GraphPad Prism 5 (GraphPad Software Inc., San Diego, USA) was used for statistical analysis and data graphing. Mean ± SD (standard deviation) was displayed for all measured biological parameters in graphs. We were used factorial ANOVA (5 × 2) flowed by post-hoc analysis using Newman–Keuls multiple comparison tests for analyzing the SMF and DOXO-induced cytotoxicity and proliferation rate, which were compared between independent variables (i.e. SMF intensity or DOXO concentration, time and cell type). We were applied factorial ANOVA (2 × 2 × 2) followed post-hoc analysis using Newman–Keuls multiple comparison tests to compared between independent variables in the ROS production, iron, and glutathione contents. Each experiment was repeated for at least three times. P-value less than 0.05 were considered as statistical significance difference.

## Electronic supplementary material

Supplementary Information
