# Supplementary material for: The Static Magnetic Field Remotely Boosts the Efficiency of Doxorubicin through Modulating ROS Behaviors
Source: Sci Rep. 2018 Jan 17;8:990. doi: 10.1038/s41598-018-19247-8 (PMC5772617; doi:10.1038/s41598-018-19247-8)
Supplement: Supplementary file 1 — Supplementary Information [file 41598_2018_19247_MOESM1_ESM.pdf]

Supplementary Information to:

The Static Magnetic Field Remotely Boosts the Efficiency of Doxorubicin through Modulating ROS Behaviors

Behnam Hajipour Verdom[1], Parviz Abdolmaleki[1,*], Mehrdad Behmanesh[2]

[1]*Department of Biophysics, Faculty of Biological Sciences, Tarbiat Modares University (TMU), Tehran, Iran.*

[2]*Department of Genetics, Faculty of Biological Sciences, Tarbiat Modares University (TMU), Tehran, Iran.*

***Corresponding Author**: Prof. Parviz Abdolmaleki; Department of Biophysics, Faculty of Biological Sciences, Tarbiat Modares University (TMU), Tehran, Iran. P.O. Box: 14115-154, Tehran, Iran. Fax: +98-21+82884717. Tel: +98 21 8288 3404. E-mail: parviz@modares.ac.ir.

Supplementary Information

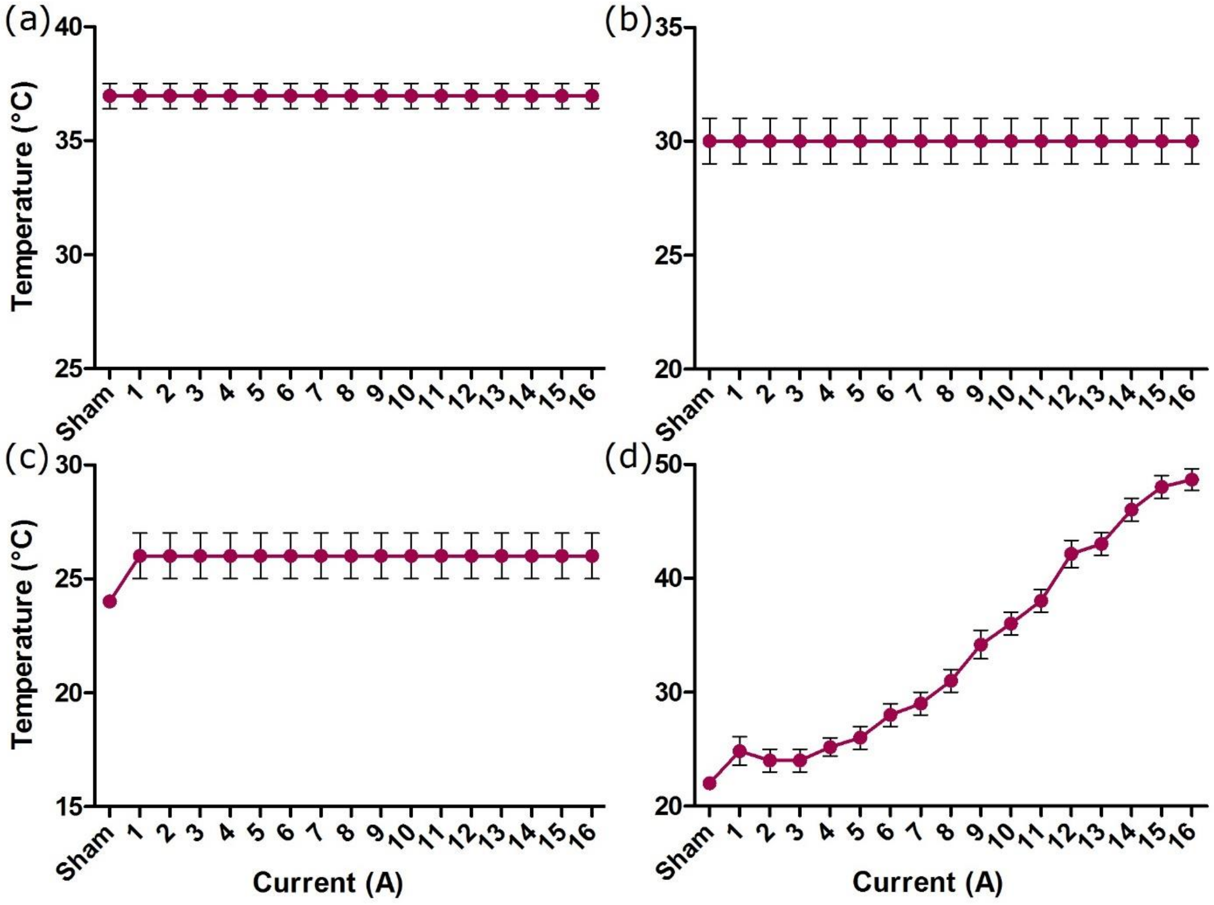

Supplementary Figure S1. The effects of applied currents of MF-exposure system on temperature of cell culture-plexiglass incubator (exposure unit). (a) Inside of exposure unit, **(b)** gap between exposure unit and iron blades, **(c)** gap between exposure unit and MF-generator coils, and **(d)** on the external of MF-generator coils. The temperatures were measured by thermometer at sham and exposure condition. Data are shown as mean ± SD (n = 3).
